# Enzyme activity effects of N-terminal His-tag attached to catalytic sub-unit of phosphoinositide-3-kinase

**DOI:** 10.1042/BSR20130075

**Published:** 2013-11-05

**Authors:** James M. J. Dickson, Woo-Jeong Lee, Peter R. Shepherd, Christina M. Buchanan

**Affiliations:** *Maurice Wilkins Centre for Molecular Biodiscovery, University of Auckland, Auckland, New Zealand; †School of Biological Sciences, University of Auckland, Auckland, New Zealand; ‡Department of Molecular Medicine and Pathology, University of Auckland, Auckland, New Zealand

**Keywords:** enzyme inhibitors, N-terminal tags, oncogenic PIK3CA, phosphatidylinositol 3-kinases, protein-serine-threonine kinases, recombinant protein production, Akt, protein kinase B, GPCRs, G-protein-coupled receptors, HTRF, homogenous time-resolved fluorescence, NT, N-terminal, NTT, N-terminal tags, PI 3-K, phosphoinositol 3-kinase, PI, phosphoinositol, rTEV, recombinant Tobacco Etch Virus protease, wt, wild-type, βic, intracellular domain of GM-CSF/IL-3 βc receptor, RAS, renin–angiotensin system

## Abstract

NTT (N-terminal tags) on the catalytic (p110) sub-unit of PI 3-K (phosphoinositol 3-kinase) have previously been shown to increase cell signalling and oncogenic transformation. Here we test the impact of an NT (N-terminal) His-tag on *in vitro* lipid and protein kinase activity of all class-1 PI 3-K isoforms and two representative oncogenic mutant forms (E545K and H1047R), in order to elucidate the mechanisms behind this elevated signalling and transformation observed *in vivo*. Our results show that an NT His-tag has no impact on lipid kinase activity as measured by enzyme titration, kinetics and inhibitor susceptibility. Conversely, the NT His-tag did result in a differential effect on protein kinase activity, further potentiating the elevated protein kinase activity of both the helical domain and catalytic domain oncogenic mutants with relation to p110 phosphorylation. All other isoforms also showed elevated p110 phosphorylation (although not statistically significant). We conclude that the previously reported increase in cell signalling and oncogenic-like transformation in response to p110 NTT is not mediated via an increase in the lipid kinase activity of PI 3-K, but may be mediated by increased p110 autophosphorylation and/or other, as yet unidentified, intracellular protein/protein interactions. We further observe that tagged recombinant protein is suitable for use in *in vitro* lipid kinase screens to identify PI 3-K inhibitors; however, we recommend that *in vivo* (including intracellular) experiments and investigations into the protein kinase activity of PI 3-K should be conducted with untagged constructs.

## INTRODUCTION

The class 1 phosphoinositide 3-kinases [PI 3-K (phosphoinositol 3-kinase)] are heterodimers consisting of an 85 kDa regulatory/adapter subunit (p85) coupled to a 110 kDa catalytic subunit (p110) with both subunits possessing several isoforms. The class I PI 3-kinases are further subdivided into two subclasses: class Ia and class Ib; the class 1a PI 3-kinases (p110α, p110β and p110δ) signal downstream of tyrosine kinases, while the single class Ib PI 3-K (p110γ) operates downstream of heterotrimeric GPCRs (G-protein-coupled receptors) [1,2]. Following receptor activation (tyrosine kinase or GPCR) the class 1 PI 3-kinases phosphorylate the hydroxyl on the 3′ position of the inositol ring in PtdIns (phosphatidylinositol) lipids principally generating the second messenger PI(3,4,5)P3. As a result the class 1 PI 3-kinases play a critical role in pathways regulating functions such as cell metabolism, cell growth and survival, cytoskeletal rearrangements and cell movement [3,4]. The class 1 PI 3-kinases also act as protein kinases [5,6], although the function of this protein kinase activity is less well defined. Furthermore, a range of oncogenic mutations have been identified in *PIK3CA* (p110α) and *PIK3R1* (p85α) [7–12] and these result in elevation of their lipid kinase activity [7,10,13] and protein kinase activity [13,14].

Owing to their importance in cell metabolism and cancer, the class 1 PI 3-kinases and oncogenic mutants have become the subjects of intense research efforts focusing on the development of a wide range of small molecule drugs to inhibit the lipid kinase activity of PI 3-K (recently reviewed in [15]). To this end many researchers are reliant upon catalytically active recombinant PI 3-K (either commercially available or produced in-house) for use in their assay systems. The majority of these recombinant kinases are produced with NTT (N-terminal tags); however, it is now recognized that NTT on p110α up-regulate the potential for oncogenic transformation of this enzyme *in vivo* and elevate downstream signalling when tagged forms of p110α are expressed in cells [16]. It appears that the molecular mechanism for this up-regulation works in part through essential Ras binding, mimicking the p110α-helical domain mutants [16] and possibly through stabilization of the catalytic subunit [17]. These findings cast doubt on the findings of studies using N-terminally tagged PI 3-K [18–21]; however, the impact of NTT on the *in vitro* activity of PI 3-K has never been determined.

We have undertaken a comprehensive study of the impact of an NT His-tag on the *in vitro* lipid kinase and protein kinase activity of all the class 1 isoforms and two major oncogenic mutants of p110α: H1047R and E545K. Two different types of assays were used to investigate lipid kinase activity: traditional autoradiography of extracted radioactive PI(3)P and HTRF (homogenous time-resolved fluorescence) analysis of PI(3,4,5)P3 levels. We also determined the IC_50_'s for several pan- and isoform-specific reference inhibitors using both His-tagged and His-tag-free PI 3-K. Here, we report that an NT His-tag has no effect on the *K*_m_ or *V*_max_, and there is no impact on *in-vitro* lipid kinase assays, or on IC_50_ determinations for the reference compounds investigated. However, it did result in a significant increase in the autophosphorylation of the catalytic subunit in oncogenic forms of p110α and elevation of autophosphorylation of all wt (wild-type) isoforms. These findings indicate that N-terminally His-tagged PI 3-K is suitable for use in *in-vitro* lipid kinase assays, and that inhibitor IC_50_ results generated using His-tagged PI 3-K are likely to be equivalent to those generated with tag-free constructs.

## MATERIALS AND METHODS

### Recombinant kinase synthesis

All class 1a isoforms and mutants were produced in-house by co-expressing full-length human p85α with the indicated human full-length catalytic subunit. Coding sequences were cloned by RT–PCR from human lymphocyte mRNA. Sf9 cells were infected with a recombinant baculovirus containing coding sequences for both the p85α (p85α; Genbank accession **NM_181523**) and p110 subunits (p110α, Genbank accession **NM_006218**; p110β, **NM_006219**; p110δ, **NM_005026**). All p110 constructs contain an N-His6 rTEV (recombinant Tobacco Etch Virus protease) tag used to purify the complex by IMAC before final purification by anion exchange on MonoQ column. The class 1b isoform was similarly produced in baculovirus-infected Sf9 cells; however, only the catalytic p110γ subunit was expressed (p110γ, **NM_002649**). The N-His6-tag removal was achieved by overnight cleavage with rTEV at 4°C, and confirmed by Western blotting of 500 ng of recombinant protein using mouse monoclonal anti-His antibody (GE Healthcare cat # 27-4710-01).

Site-directed mutagenesis of p110α to yield the oncogenic mutants was performed by using either complementary (overlapping sense and antisense) oligonucleotides containing sequence mismatches incorporating the desired point mutation, or back-to-back phosphorylated primers spanning the region to be mutated (with one primer containing the desired point mutation). Whole plasmid PCR reactions were performed using a high-fidelity DNA polymerase (Stratagene Pfu Ultra II Fusion HS) and the previously cloned wt p110α catalytic coding sequence as the template. Following PCR amplification of mutated sequences, the template DNA was removed by digestion with DpnI restriction endonuclease. In mutagenesis reactions using overlapping primers, the mutated plasmid was recovered by direct transformation into DH5alpha cells. For reactions using phosphorylated primers following removal of template DNA with DpnI, the (mutated) PCR products were self-ligated with T4 DNA ligase prior to transformation into DH5α cells. For both the methods, resultant plasmids were sequenced to confirm the insertion of the desired mutations prior to generation of recombinant baculovirus.

### Recombinant βic (intracellular domain of GM-CSF/IL-3 βc receptor) production

Production and purification of the His-tagged recombinant βic protein encompassing amino acids 445–881 of the βic has been previously described in [22,23].

### Inhibitors

Wortmannin and LY294002 were from Sigma-Aldrich; TGX-221 was from Symansis; PIK-75, A66 and AS252424 were synthesized in-house as previously described [23,24].

### Lipid kinase assays

#### TLC/autoradiography using PI (phosphoinositol) as substrate

For one TLC plate (consisting 12 points/plate), kinase (0.3 μg for p110α, δ, H1047R and E545K; 0.65 μg for p110β; 1.62 μg for p110γ) was made up to 260 μl in buffer containing 40 mM Tris/HCl, 200 mM NaCl, 1 mM EDTA (pH 7.4). Drugs were dissolved in DMSO and serially diluted in the same, before addition of 0.6 μl into a clean microcentrifuge tube. Each reaction point incorporates 20 μl of kinase mixed with 10 μl of 1 mg/ml PI (Lipid Products) in 10 mM Tris/HCl, 1 mM EDTA (pH 7.4), 30 μl of ATP mix (10 mM MgCl_2_, 200 μM ATP, 1 μCi γ^33^P-ATP) and 0.6 μl of 100× inhibitor or DMSO (final ATP concentration of 100 μM). The reaction is incubated for 1 h at room temperature (20°C) and stopped with 100 μl of 1 M HCl. Lipids were extracted by adding 200 μl of chloroform/methanol (1:1, v/v), vortexing, centrifuging (9000 ***g*** for 2 min) and removing 200 μl of the upper phase. Lipids were then re-extracted by adding 80 μl of methanol/1 M HCl (1:1, v/v) before vortexing, centrifuging (9000 ***g*** for 2 min) and complete removal of the upper phase. The extracted lipids were dried (speed-vac for 40 min) and resuspended in 30 μl of chloroform: methanol (4:1, v/v) by vortexing. TLC plates were pre-treated with a solution containing 8 mM oxalic acid and 1 mM EDTA (pH 8) in MQ H_2_O/ethanol (3:1, v/v), and allowed it to dry at room temperature overnight. Lipids were separated on the TLC plates using propan-1-ol/glacial acetic acid/MQ H_2_O (65:4:31, v/v). Assay results were analysed by autoradiography (Molecular Dynamics Storm 680 PhosphorImager and quantified using ImageQuantTL software).

#### HTRF using PI(4,5)P2 as substrate

Enzyme titrations and IC_50_ values were determined using the PI 3-K (human) HTRF Assay (Millipore, #33-016) according to the manufacturer's instructions with 10 μM ATP. All PI 3-K isoforms were made in-house as described above and used in the range of their EC_65–80_ titration for inhibitor studies (18 ng/ml for H1047R, 6.5 ng/ml for E545K, 50 ng/ml for p110α, 400 ng/ml for p110β, 65 ng/ml for p110δ and 400 ng/ml for p110γ). Drugs were dissolved in DMSO and serially diluted in the same. Final DMSO concentration in assay was 2.5%.

#### Kinetic analysis

Kinetic analysis was undertaken using the PI 3-K HTRF assay (above), with four replicates per data point for 3 time points (0, 5 and 10 min) per experiment. ATP was made up in 1× reaction buffer (provided in the kit) to final concentrations of 10, 20, 50, 100 and 300 μM. PI(4,5)P2/kinase mix was made up according to the manufacturer's instructions (along with PI(4,5)P2 substrate only for minus enzyme controls). Quadruplicate 5 μl volumes of ATP at prepared concentrations were added to a 384 well plate. Stop solution (5 μl) was added to the 0 min time point wells, and the addition of 15 μl of PI(4,5)P2/kinase mix was staggered so that the 5 and 10 min reactions could all be terminated by the addition of Stop solution to all wells at the same time. Detection mix (5 μl) was added to all wells and the plate was read according to manufacturer's instructions 3 h later (Biotek Synergy 2).

### Protein kinase assays

Unless otherwise stated, protein kinase assays were carried out in a buffer containing 5 mM MgCl_2,_ 50 mM NaCl, 20 mM Tris/HCl (pH 7.4), 0.1 mM Na-orthovanadate, 12 μM ATP, 5 mM DTT (dithiothreitol) and 2 μCi γ ^33^P-ATP; Each reaction tube contained 0.5 μg kinase, 0.5 μg βic and inhibitors at stated concentrations. Unless otherwise stated, incubations were allowed to proceed for 20 min at 32°C and terminated by the addition of 5× electrophoresis sample buffer before complete denaturation at 99°C for 5 min. Components were separated by SDS/PAGE before the gels were stained with Coomassie Brilliant Blue, dried and analysed by autoradiography (Molecular Dynamics Storm 680 PhosphorImager and quantified using ImageQuantTL software).

## RESULTS

### Impact of NT His-tag on lipid kinase activity

**Figure 1:**
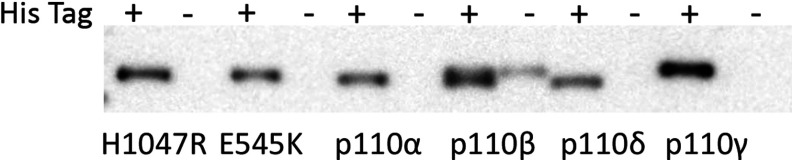
Confirmation of His-tag removal from PI 3-K isoforms ascertained by Western blot using Anti His-tag antibody

After proving the removal of His-tag via Western blotting (Figure 1), we investigated the impact of an NT His-tag on several aspects of two *in vitro* lipid kinase assays using the four class 1 isoforms and two oncogenic p110α mutants (H1047R and E545K). Specifically, we undertook studies into enzyme kinetics using PI(4,5)P2 as a substrate and measured PI(3,4,5)P3 by HTRF; we further used the HTRF assay to determine any differences in enzyme activity as determined by titration of the kinases. Finally, we used both HTRF (with PI(4,5)P2 as substrate) and TLC/autoradiography (with PI as substrate) to determine if there was any difference in inhibitor IC_50_ using the different forms of the enzyme.

**Figure 2:**
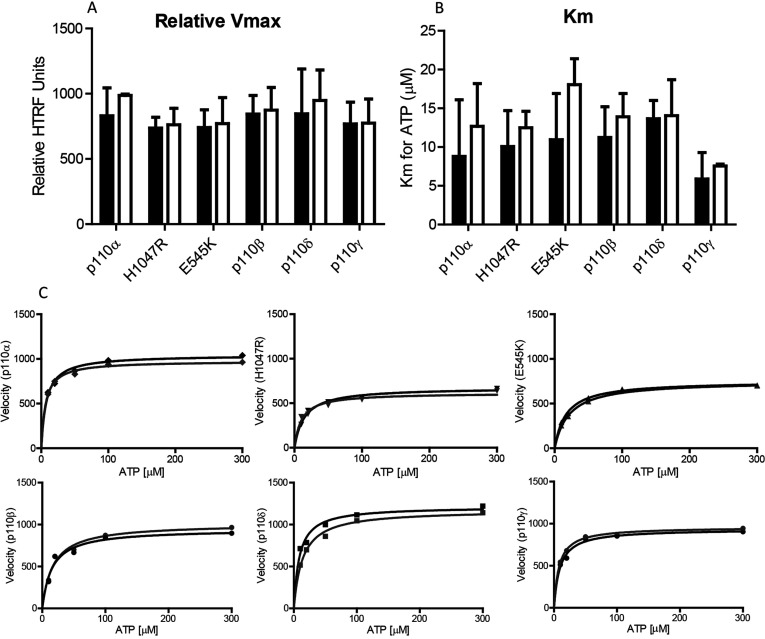
Kinetic analysis of His-tag (solid bars) and His-tag-free (open bars) kinases as labelled (median ± range, *n*≥2) (**A**) Relative *V*_max_, (**B**) *K*_m_ for ATP in μM, (**C**) Representative Michaelis–Menten plots for all kinases as detailed on *y*-axis (His-tag, black line; His-tag-free, grey line).

Our investigation into the kinetics of the enzyme reaction clearly showed that there was no difference in *V*_max_ or *K*_m_ between His-tagged and tag-free forms of the various enzymes (Figures 2 (A) and 2(B)).

**Figure 3:**
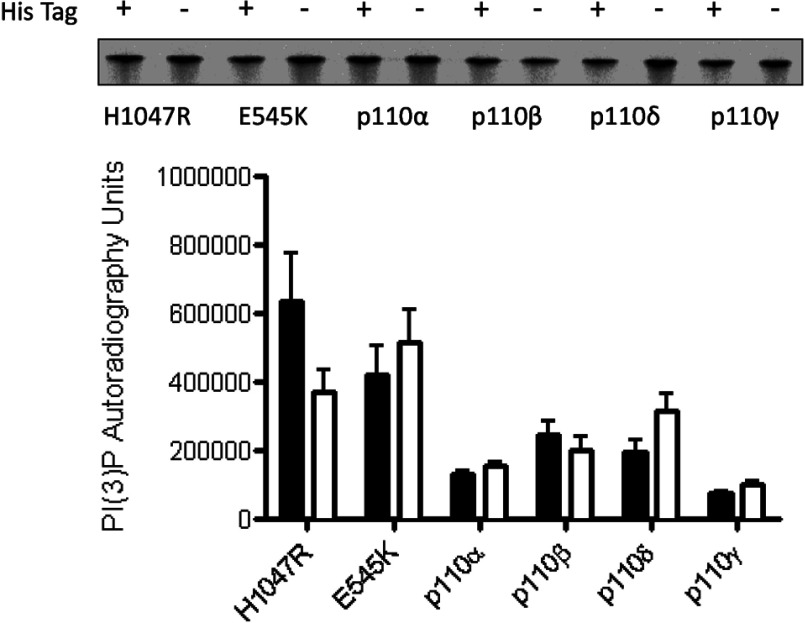
Effects of His-tag on lipid kinase activity as measured by generation of PI(3)P in the radioactive TLC assay Representative autoradiograph and quantitation; His-tag (solid bars) and His-tag-free (open bars) kinases as labelled (median±S.E.M., *n*≥3).

Enzyme titration curves for all the class 1 isoforms and the oncogenic mutants showed no tag-dependent difference in the effective concentration of the kinases (Table 1), although the oncogenic mutants were more active for a given concentration than the wt p110α as observed previously [25,26]. This was confirmed by a side-by-side comparison of lipid kinase activity using TLC/autoradiography (Figure 3). Furthermore, our investigations comparing the effect of the His-tag on the IC_50_'s of isoform-specific small molecule inhibitors proved that the presence of the NT His-tag had no impact on compound IC_50_ (as measured by two different assay types using two different inositol phosphate substrates (Tables 2A and 2B).

**Table 1:** HTRF enzyme titration results (in ng/ml) for different isoforms ± His-tag

	^p110α^		^p110α H1047R^		^p110α E545K^		^p110β^		^p110δ^		^p110γ^	
His-tag	**+**	**−**	**+**	**−**	**+**	**−**	**+**	**−**	**+**	**−**	**+**	**−**
EC_60_	80	64	6	9	20	20	171	242	56	75	311	234
EC_85_	498	403	57	47	60	58	402	661	391	342	619	521

**Table 2:** IC_50_s (in nM) of known isoform specific PI 3-K inhibitors against different isoforms ± His-tag screened in (A) radioactive TLC lipid kinase assay using 100 μM ATP, and (B) HTRF assay using 10 μM ATP

(A) Radioactive TLC lipid kinase assay using 100 μM ATP												
Radioactive TLC assay	^p110α^		^p110α H1047R^		^p110α E545K^		^p110β^		^p110δ^		^p110γ^	
His-tag	**+**	**−**	**+**	**−**	**+**	**−**	**+**	**−**	**+**	**−**	**+**	**−**
A66	9.1	7.7	7.2	7.4	5.6	4.2						
PIK-75	2.9	2.8	2.1	1.5	1.7	1.6					11	15
TGX221							2.8	3.2				
PIK108							2.4	2.3				
IC87114									22	20		
CAL101									1.9	1.5		
AS252424											49	70
(B) HTRF assay using 10 μM ATP												
HTRF assay	^p110α^		^p110α H1047R^		^p110α E545K^		^p110β^		^p110δ^		^p110γ^	

His-tag	**+**	**−**	**+**	**−**	**+**	**−**	**+**	**−**	**+**	**−**	**+**	**−**
A66	16	28	21	22	9.1	6.4						
PIK-75	0.9	1.4	1.1	1.0	0.8	1.0					44	26
TGX221							15	11				
PIK108							14	14				
IC87114									247	293		
CAL101									13	13		
AS252424											94	53

## IMPACT OF NT HIS-TAG ON PROTEIN KINASE ACTIVITY

In addition to lipid kinase activity, PI 3-K exhibits protein kinase activity, with an ability to auto-phosphorylate its own sub-units [5,6] as well as other protein substrates [27–33]. The effect, if any, of an NT His-tag on this protein kinase activity has never been explored, so we undertook a series of experiments to probe the impact of the NT His-tag on both autophosphorylation and on the phosphorylation of an exogenous substrate (an intracellular fragment of the GM-CSF/IL-3 βc receptor (βic) [14,33].

**Figure 4:**
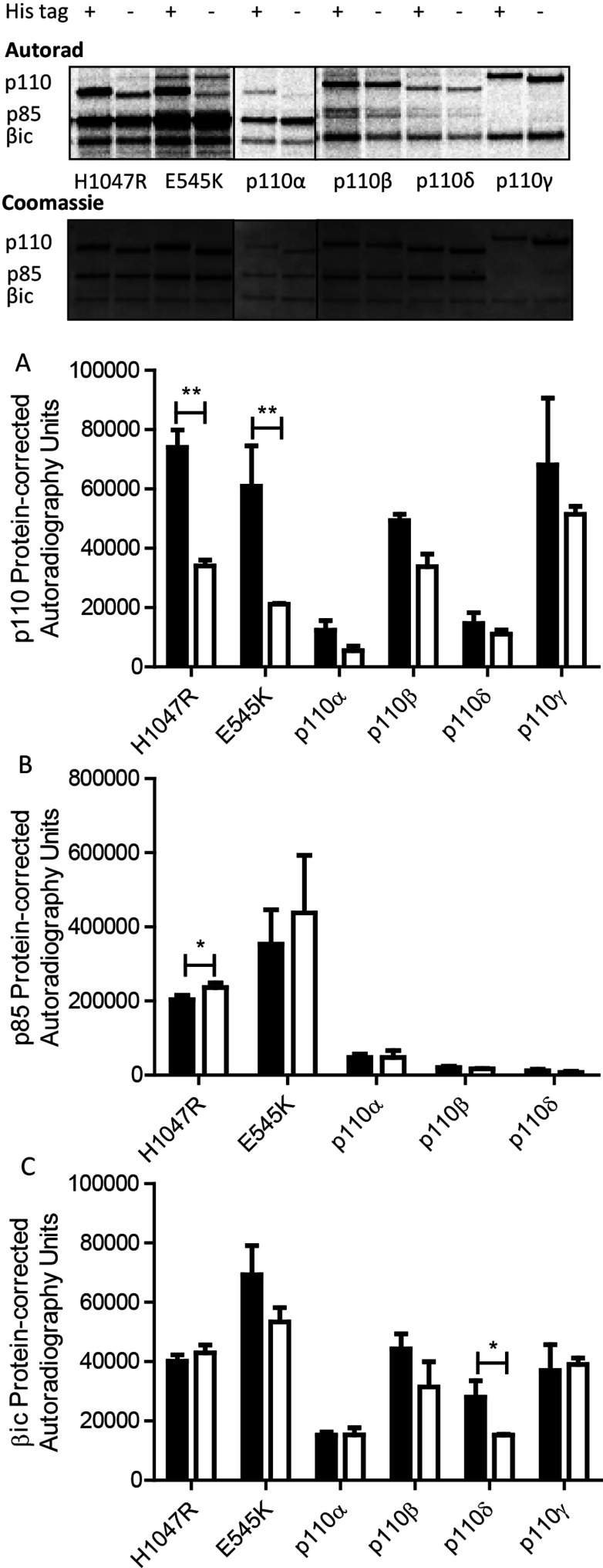
Effects of His-tag on protein kinase activity as measured γ^33^P incorporation into proteins Representative autoradiograph and gel stained with Coomassie Brilliant Blue are shown, band shifts obvious for p110 are because of the presence or absence of His-tag; Quantitation of His-tag (solid bars) and His-tag-free (open bars) kinases as labelled for phosphorylation of (A) p110, B) p85 (all isoforms except p110γ) and (C) βic (median ± S.E.M., *n*≥2), asterisks indicate the statistical significance as shown **P<0.01, *P<0.05. Autoradiography units corrected for protein loading based on Coomassie Brilliant Blue staining intensity.

The addition of the NT His-tag had marked effects on the oncogenic mutants’ p110 autophosphorylation, with both mutants exhibiting a significant increase in p110 phosphorylation with His-tag incorporation (Figure 4 A). The His-tagged PI 3-Kα-wt, β, δ and γ also showed elevated p110 phosphorylation (although not statistically significant; Figure 4A). The incorporation of the NT His-tag had no effect on p85 phosphorylation (Figure 4B); however, some variations were observed for βic phosphorylation (Figure 4C), with PI 3-Kδ exhibiting elevated βic phosphorylation with His-tag incorporation.

## DISCUSSION

A recent paper by Sun et al. [16] presented evidence that NTT on p110α activates the potential for oncogenic transformation of this enzyme *in vivo* and elevates downstream signalling when tagged forms of p110α are expressed in cells. All the NTT constructs tested induced oncogenic transformation whether they were a His-tag, flag-tag or a myristylation signal. These observations built on the original study of Yu et al. [17] who also found that a series of different NTT [myc, Tris-HA and GST (glutathione transferases)] resulted in increased stability and lipid kinase activity for p110 monomers. These researchers further observed that NTT and p85 binding were complementary not synergistic, suggesting that the NTT substitutes for the p85 sub-unit, which was also thought to bind at the N-terminus of p110 [17]. Taken together, these data indicate that it is probable that any NT addition will have an activating effect; however, the mechanisms by which this activation was effected were unknown. Here for the first time, we have undertaken a targeted functional study to examine the *in vitro* effects of an NT His-tag on the lipid and protein kinase activity in order to elucidate the probable mechanism of action. We investigated all the class 1 PI 3-K isoforms (consisting of p110/p85 for the PI 3-Kα, -β, -δ and p110 only for PI 3-Kγ) and two PI 3-Kα oncogenic mutants (the H1047R catalytic domain mutant and the E545K helical domain mutant). Our results show that the incorporation of an NT His-tag has no effect on lipid kinase activity as measured by enzyme kinetics and enzyme titration, indicating that the increase in oncogenic transformation and elevated signalling through the Akt (protein kinase B or PKB) pathway previously observed [16] is not likely to be because of increased PI lipid phosphorylation. We also proved that the presence/absence of NT His-tag has no impact on inhibitor IC_50_ determination (Tables 2A and 2B), which is an important consideration for the vast number of laboratories using recombinantly produced PI 3-K for screening lipid kinase inhibitors. In contrast to the lack of impact on lipid kinase activity, we were interested to find that NT His-tags do impact the protein kinase activity. More specifically, the NTT resulted in elevated p110 autophosphorylation for the oncogenic mutants (and wt isoforms to a lesser extent), as well as increased phosphorylation of βic by p110δ. There was little to no variation in effect observed in relation to p85 phosphorylation.

In summary, we conclude that the increased oncogenic transformation and Akt signalling associated with NTT PI 3-K in cells is not mediated through an intrinsic elevation in lipid kinase activity. Sun et al. [16] concluded that any NTT would have an activating effect via inducing a conformational change in p110α similar to that of helical domain mutations, thus disrupting inhibition by the regulatory subunit, p85. Our findings indicate that this suggested conformational change if it occurs, has no impact on lipid kinase activity. Furthermore, if this was true then we would expect to see a difference between the helical and catalytic domain mutants. Although no difference was seen in our lipid kinase assay, there are some perturbations of the protein kinase activity, impacting both the oncogenic mutants, and the wt isoforms to a lesser degree. Other intracellular protein/protein interactions [e.g. PI 3-K/RAS (renin–angiotensin system) interactions] have yet to be investigated, and could provide insight into the mechanism, since it has also been observed that interaction with p85 is not essential for the oncogenic activity mediated via NTT-p110α, but interaction with RAS is [16].

From this and other studies we can make the following conclusions and recommendations; *in vivo* (including intracellular) experiments and investigations into the protein kinase activity of PI 3-K should ideally be conducted with untagged constructs, whereas it is safe to carry out *in vitro* lipid kinase screening using tagged recombinant protein.

The findings reported from this investigation act to remind researchers using recombinantly produced proteins to be aware that the inclusion of various tags commonly incorporated to aid purification, can impact the functional activity of the protein.
